# Use of a flowable haemostat versus an oxidised regenerated cellulose agent in primary elective cardiac surgery: economic impact from a UK healthcare perspective

**DOI:** 10.1186/s13019-017-0660-y

**Published:** 2017-11-29

**Authors:** Mayur R. Joshi, Jacqueline Latham, Gabriel Okorogheye

**Affiliations:** Baxter Healthcare Ltd., Wallingford Rd, Compton, Newbury RG20 7QW UK

**Keywords:** Floseal, Economic impact, Cardiac surgery

## Abstract

**Background:**

Flowable haemostatic agents have been shown to be superior to non-flowable agents in terms of haemostatic control and need for transfusion products in patients undergoing cardiac surgery. We investigated the economic impact of the use of a flowable haemostatic agent (Floseal) compared with non-flowable oxidised regenerated cellulose (ORC) agent in primary elective cardiac surgery from the perspective of the UK National Health Service (NHS).

**Methods:**

A cost-consequence framework based upon clinical data from a prospective trial and an observational trial and NHS-specific actual reference costs (2016) was developed to compare the economic impact of Floseal with that of ORC. The individual domains of care investigated comprised complications (major and minor) avoided, operating room time savings, surgical revisions for bleeding avoided and transfusions avoided. The cost impact of Floseal versus ORC on ICU days and extended bed days avoided was modelled separately.

**Results:**

Compared with ORC, the use of Floseal would be associated with overall net savings to the NHS of £178,283 per 100 cardiac surgery patients who experience intraoperative bleeding requiring haemostatic therapy. Cost savings were apparent in all individual domains of care (complications avoided: £83,536; operating room time saved: £63,969; surgical revisions avoided: £34,038; and blood transfusions avoided: £22,317). Cost savings per 100 patients with Floseal over ORC in terms of ICU days avoided (*n* = 30) and extended bed days avoided (*n* = 51.7) were £57,960 and £21,965, respectively. A sensitivity analysis indicated that these findings remained robust when the model parameters representing the clinical benefit of Floseal over ORC were reduced by up to 20%.

**Conclusions:**

Despite higher initial acquisition costs, the use of flowable haemostatic agents achieves substantial cost savings over non-flowable agents in cardiac surgery. These cost savings commence during the operating theatre and appear to continue to be realised throughout the postoperative period.

**Electronic supplementary material:**

The online version of this article (10.1186/s13019-017-0660-y) contains supplementary material, which is available to authorized users.

## Background

Elective cardiac surgery accounted for £300 million of healthcare costs in the UK in 2015–2016, representing 5.5% of all elective inpatient hospital costs annually [1]. Patients undergoing these procedures spent a total of 147,943 days in hospital or 3.8% of all bed occupancy following elective inpatient admission [1]. Major determinants of prolonged postoperative inpatient stay include excessive blood loss and bleeding-related complications [2, 3]. Patients undergoing cardiac surgery are at particular risk of such events due to the nature of surgical procedures which may require vascular anastomoses or repair of iatrogenic breaches in the cardiac wall and major vessels. Furthermore the risk of coagulopathy and bleeding is increased by the use of mechanical surgical adjuncts such as cardiopulmonary bypass and therapeutic interventions such as heparinisation [4–6].

Haemostatic agents have been used in clinical practice since as early as 1909 [7] and a variety of different agents now exist including fibrin and gelatin based products, glues and gels. Non-flowable haemostatic patches of oxidised regenerated cellulose (ORC) or absorbable gelatin (SURGICEL NU-KNIT®,^1^ Ethicon–Johnson & Johnson, Somerville, NJ, and Gelfoam®, Pfizer, New York, NY; not approved for use in Europe) can arrest bleeding when applied directly to the site of the bleed but are less effective at surgical sites that are aggressively bleeding or difficult to reach [8]. Floseal (Baxter Healthcare Corporation, Deerfield IL, USA) is a flowable haemostatic agent that was developed to overcome the limitations of non-flowable haemostatic agents [9]. Floseal is indicated in the European Union in surgical procedures as an adjunct to haemostasis when control of bleeding, ranging from oozing to spurting, by ligature or conventional procedures is ineffective or impractical. Floseal was shown to be superior to non-flowable haemostatic agents in terms of successful termination of operative bleeding, time to haemostasis, rates of postoperative bleeding and rates of transfusion product use in a prospective randomised controlled trial [8]. A subsequent investigation of hospital economic impact showed that the better efficacy of Floseal over non-flowable agents translated into cost savings when used on a routine basis in cardiac surgery [10]. The Tackett study used a health economic model that applied United States (US) healthcare costs to the results of a large clinical trial [8]. Therefore its conclusions may be less applicable to the costs associated with cardiac surgery in the United Kingdom (UK).

Another approach for considering the clinical and economic impact of haemostatic agents is to evaluate the influence of different agents on postoperative hospital stay. A retrospective claims database study analysed the length of hospital stay (LOS) in patients who underwent cardiac surgery in the USA [11]. This study considered the likelihood of patients requiring extended inpatient care compared with the expected duration demonstrating a significantly lower odds ratio (OR) for extended LOS in patients treated with Floseal (OR = 0.791, *p* < 0.01) compared with other treatments for haemostasis. The incidence of a longer than expected hospital stay was also significantly lower in the Floseal-treated group compared with other therapies. However, while supporting the contention that the use of the flowable haemostatic matrix delivered benefits beyond the operative period, OR data indicate the likelihood of exceeding the expected length of stay and thus cannot be directly quantified in terms of saved bed days and saved costs.

Ideally, the marginal costs associated with different healthcare interventions would be evaluated in a prospective randomised clinical study. However, such a study of cardiac surgery outcomes in an individual country would require considerable time to conduct and may not yield more valuable insights than those available from the results of existing research. We postulated that appropriate modelling of the cost implications to the National Health Service (NHS) of treatment with Floseal could be based upon the principles established previously [8, 10] and augmented by the inclusion of additional clinical data relevant to practice within the UK. Furthermore, since the characteristics of healthcare in the UK will impact the outcomes delivered by it, we proposed that the incorporation of new, UK-specific information would yield a more comprehensive and representative compelling evaluation. We therefore developed an updated model based on UK-specific healthcare data and costs that also addressed the effect of Floseal on length of stay.

## Methods

### Model design

We developed a cost-consequence framework based upon the work of Tackett et al. [10] to compare the economic impact of Floseal with that of ORC using data from an observational clinical trial [11] and a prospective clinical trial [8].

In addition we developed a supplementary model module to evaluate the specific direct costs of prolonged intensive care unit (ICU) stay and excess bed stay costs. We analysed bed occupancy costs separately to avoid double-counting, as bed occupancy costs are already included in the overall costs of inpatient health care.

### Clinical inputs

The majority of clinical inputs used in the model have been described previously [8, 10]. In brief, patients contributing data were scheduled for elective primary cardiac or thoracic surgery and had been subsequently randomised to treatment with either Floseal or one of two comparator topical haemostatic agents available on the research institution’s hospital formulary in case of an intraoperative bleed [8]. The study participants had provided written informed consent and the protocol was approved by the local ethics committee. For those patients who had intraoperative bleeding, related clinical outcomes and interventions were collected: haemostasis time in minutes, length of ICU stay in days, the incidences of transfusion of blood products, surgical revision for bleeding, and major and minor postoperative complications. Major postoperative complications comprised stroke, shock, sepsis and myocardial infarction. Minor postoperative complications were defined as renal failure, respiratory insufficiency and inotropic support lasting more than 24 h. All clinical outcomes were statistically significantly in favour of Floseal versus the comparator with the exception of major complications (*p* = 0.34) and ICU stay (*p* = 0.1) which did not differ between study arms. The percentage breakdown of patients experiencing each type of major and minor postoperative complication included in the model was provided by the original study investigators [8]. Finally, the overall impact of Floseal upon hospital bed occupancy was inferred from published data [1] which summarised historical extended LOS for elective hospital inpatients according to specific complications and comorbidities. Only excess bed days for major complications were included in this calculation to avoid the potential for double counting. This was because it was considered that minor complications would either occur concurrently with major complications or otherwise be unlikely to extend hospital stay.

### Cost inputs

Cost inputs for the model fell into one of three categories: resource use, initial surgery, and complications. Since the data sources used for each element of the model were each derived from actual healthcare costs within the year prior to analysis, no adjustment for inflation was necessary. The sources of the majority of cost inputs used in the model were NHS reference publications.

Resource use was determined to have four elements: acquisition costs for haemostatic therapy, blood transfusions, number of transfusion units, and operating theatre time. The costs of Floseal and comparator haemostats were taken from the NHS list of prices for providers [12]. One treatment (Gelfoam, Pfizer, New York NY, USA) that was included in the previously published model was excluded as this agent is not available in the UK. The number of kits used for an average case in the UK was derived from an independent market research study completed in 2015 and funded by Baxter Healthcare Ltd. (see Additional file 1). The cost of an individual unit of blood for transfusion was accessed from the National Institute of Health and Care Excellence [13] and the number of units transfused was derived from the prior clinical research [8]. Haemostasis time was defined as the time from removal of the cardiopulmonary bypass cannulae to closure of the sternum, as defined previously [8] and was used to represent the operating theatre time saved. The economic impact of differences in operating theatre time was calculated by multiplying the time by the mean cost of running cardiac surgery theatres in four different hospital trusts [14].

The initial cost of surgery without complications was separated into i) ‘uneventful’ surgery, i.e. with neither complications nor need for revision to address postoperative bleeding, and ii) surgery without complications but which did require revision. The mean cost to the NHS of ‘uneventful’ cardiac surgeries was calculated as the weighted mean of unit costs for all cardiac procedures according to comorbidity and complexity score, multiplied by the number of individual procedures conducted in during the reporting period 2015–2016 [1]. Since no data were available to estimate the cost of the of surgery with subsequent revision, this was calculated as the mean cardiac surgical cost plus the mean percentage incremental cost (30%) of moving from one comorbidity and complication (CC) subcategory to the next level of severity for all procedures under consideration. CC scores are used in reporting of NHS costs according to Health Resource Groups (HRG) and are calculated on the basis of 2 points for a major condition and 1 point for a minor condition; subcategories cover a 3 point range for most conditions. Hence the occurrence of a major complication is likely to move a patient to the level above.

The incremental economic impact of postoperative complications was inferred from published Department of Health NHS Reference Costs for 2015 to 2016 [1]. A weighted mean cost was calculated on the basis of the average cost for each subcategory of the individual HRG codes for stroke (AA35A–E), heart failure or shock (EB03A–D), sepsis (WJ06A,B,D,E,G,H), and myocardial infarction (EB10A-D) multiplied by the number of hospital spells for each subcategory. Postoperative cardiac surgery patients were assumed to have CC scores above the lowest severity and cost category for each major complication of interest (AA35F, EB03E, WJ06C,FJ and EB10E) and these were excluded in the average cost calculation to avoid potential underestimation of costs likely to be incurred. A similar approach was used to calculate the economic impact of minor complications with the HRG codes for acute kidney injury (c.f. renal failure; LA07J,K,M,N), respiratory failure (DZ27N,Q,R,T,U) and heart failure or shock (EB03B–D) in place of inotropic support >24 h. In this case, to mitigate the potential for over estimation of costs where minor complications occurred concomitantly with each other or major complications in this postoperative population, the highest cost categories (LA07H, LA07L, DZ27M,P,S and EB03A) were excluded from average cost calculation.

The cost of hospital care in the ICU and for excess in patient days during an extended LOS were derived from national NHS publications [1, 15]. For excess bed days this way calculated as the mean of the reported daily cost of excess bed days for elective admissions for cardiac surgery.

The input values used in the model are summarised in Table 1.

**Table 1:** Model cost inputs

Parameter	Cost (GBP)	Range for sensitivity (%)
Main model components		
Operating theatre (per 15 min)	£396.5	−15% to +25%
Cardiac surgery (without complications)		
Without revision	£12,679	±15%
With revision	£16,461	±15%
Cardiac surgery with major complications		
Stroke	£25,206	±20%
Shock	£18,529	±20%
Sepsis	£20,199	±20%
Myocardial infarction	£16,535	±20%
Any combination of above major complications	£22,129	±20%
Cardiac surgery with minor complications		
Renal failure	£17,558	±20%
Respiratory insufficiency	£14,150	±20%
Inotropic support >24 h	£18,197	±20%
Any combination of above minor complications	£16,312	±20%
Incremental cost of a single unit allogenic blood transfusion	£164	±10%
Average number of units of blood transfused per surgical case	4.2	±10%
Difference in haemostatic agent cost per case	£14.43	±25%^a^
Supplementary model data		
Cost of Intensive care for 1 day	£1932	NA
Cost of an extended length of stay bed day	£425	NA

^a^variation in number of units used at fixed cost per unit

### Model analyses

The model construct allowed calculation of the economic and clinical impact resulting from the use of Floseal as an alternative to ORC in cardiac surgery. Results are presented in terms of outcomes avoided on an annual basis assuming treatment of 100 patients who experience intraoperative bleeding requiring haemostatic therapy.

In addition to the principal analysis based upon the previously observed clinical trial data and assumptions based on national UK data, an additional analysis was completed to explore the sensitivity of the model to changes in input cost values. The inputs for this sensitivity analysis were consistent with the inherent variability of costs across different healthcare institutions in the UK, for example the hourly running cost of cardiac surgery theatres varied from £1009 per hour to £1511 per hour (−15% to +27.5%) [14]. The upper end of this range is consistent with the hourly cost of running an operating theatre for trauma in England (£1486) reported by Ang et al. [16]. The ranges used in the sensitivity analysis are summarised in Table 1.

## Results

### Main model

**Fig. 1:**
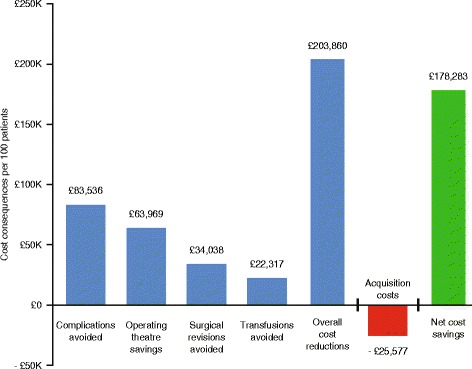
Domain specific and overall cost consequences per 100 treated patients

Application of NHS costs to the principal model indicates that the incremental cost of Floseal over ORC per 100 patients (£25,577) resulted in an overall reduction in costs associated with elective cardiac surgery of £203,860. This translated into a net cost saving of £178,283, or approximately £1783 per patient. The use of Floseal was associated with cost savings over ORC in all individual domains of care (Fig. 1). Considering these individual domains in isolation, the incremental cost of Floseal was more than offset by cost savings in terms of complications avoided (*n* = 18.2; £83,536), operating room time (40.3 h; £63,969), and avoided surgical revisions for bleeding (*n* = 9; £34,038). Moreover, the additional cost of Floseal based on 100 patients was almost completely (87%) offset by savings in the cost of blood transfusions alone (*n* = 32.4; £22,317).

### Supplementary model

The supplementary model demonstrated that the use of Floseal over ORC was associated with 30 fewer ICU days and 51.7 fewer extended bed days per 100 patients (Table 2). Cost savings per 100 patients in terms of ICU days avoided (£57,960) were more than twice the incremental cost of Floseal treatment over ORC (Table 2); while the value of excess bed stay days avoided (£21,965) was close to the additional acquisition cost of Floseal.

**Table 2:** Estimated annualised impact of using Floseal in 100 procedures

Outcome	Clinical	Economic (Associated cost savings)
Acquisition costs		
Incremental cost of Floseal use		£(25,577)
Main model		
Complications		
Number of complications avoided (n)	18.2	£83,536
Operating theatre		
Cumulative theatre time saved (hours)	40.3	£63,969
Surgical revision		
No of surgical revisions for bleeding avoided (n)	9.0	£34,038
Transfusions		
No of transfusions avoided	32.4	£22,317
Overall cost reduction	–	£203,860
Supplementary model		
Hospital bed savings		
Number of ICU days avoided	30.0	£57,960
Number of extended stay bed days avoided	51.7	£21,965

### Sensitivity analysis

**Fig. 2:**
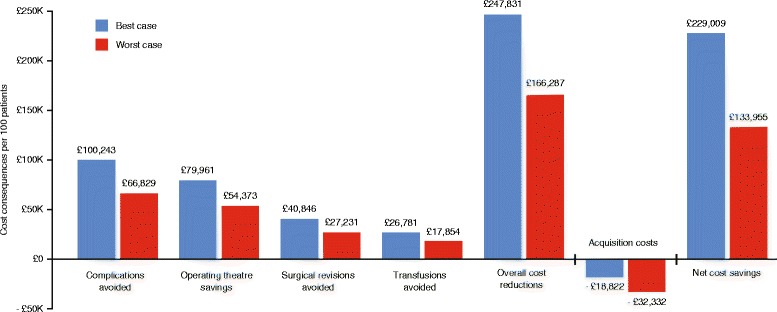
Sensitivity to changes in model assumptions. This figure uses the ranges of outcomes shown in Table 1 as a basis for sensitivity

Figure 2 shows the results of the sensitivity analysis that explored the influence of changes in the model input cost values. The cost ranges investigated in this analysis are shown in Table 1. The results indicate that cost savings associated with Floseal use persist even when the notional economic impact of individual adverse clinical outcomes is reduced by as much as 20% (Fig. 2). The overall cost reductions per 100 patients for all domains under consideration ranged from £166,287 (worst case impact) to £247,831 (best case impact) which translated into net cost savings of £133,955 and £229,009, respectively, after taking into account the incremental costs of Floseal over ORC.

## Discussion

Topical haemostatic agents are a mainstay of operative care not only in cardiac and vascular surgery but also in orthopaedic, spinal, thyroid and abdominal surgery. We sought to apply specific UK costs data to the outcomes of a study in cardiac surgery patients to ascertain the magnitude of any costs savings associated with the use of a flowable haemostatic matrix compared to an alternative (ORC) also available in the UK.

Our model indicates economic benefits associated with the choice of Floseal over ORC across all four domains evaluated (Fig. 1). The individual cost savings in three of the domains alone (complications avoided, operating theatre savings, and surgical revisions avoided) exceeded the incremental acquisition costs associated with Floseal. This analysis excluded the impact of non-blood transfusions e.g. fresh frozen plasma platelets which were reported to be reduced in the treatment arms randomised to Floseal versus the comparator but for which no separate analysis according to treatment received was reported [8]. In order to ascertain the robustness of our model to changes in the underlying assumptions we stress tested it using real cost inputs that would minimise the capacity of the model to yield outcomes in favour of Floseal (Fig. 2). In all instances the cost benefit of treatment with Floseal persisted overall and in each of the domains considered.

The observations in favour of Floseal reported by the principal model are supported by those for the supplementary model that also considered bed day costs (Table 2). A reduction in overall LOS due to the prevention of major complications was demonstrated in favour of Floseal. These savings in terms of ICU bed days might contribute to increased patient throughput in terms of number of operations that can be performed annually.

We estimated the costs of surgery and care for both major and minor complications on the basis of NHS HRG data for the most recent year available (2016). Unlike the equivalent health care costs calculated previously for the US [10], based on ICD-9-CM procedure codes, HRG currency codes are based upon primary diagnoses and subcategorised according to the level of concomitant illness experienced by the patient. Data are then presented for different populations based upon the nature of their admission to hospital, such as day case, elective inpatient and non-elective inpatient. While the sub-population of elective inpatients matches precisely the admission status of our intended area of study, it was necessary to infer certain cost aspects from the source data.

The estimate of the cost of a cardiac surgery admission with revisions assumed that the patients requiring these would incur care costs associated with one level higher CC severity in the HRG codes for each procedure type. This approach may under estimate the incurred costs as the costs associated with a revision may be closer to those of a new operation. However, this approach was considered to be conservative and was used in place of the full cost of a procedure. In some patients this approach may result in an overestimate of the incremental cost of revisions since some patients might already be in the highest category of cost whilst others may not have had sufficiently large a change in their CC score to move them into the upper cost category. This is justifiable, since the categories themselves have arbitrary boundaries and in reality the increase in costs with increased CC score will be a continuum.

In the randomised trial from which clinical data were obtained, Nasso et al. considered inotropic support lasting more than 24 h to be a minor complication [8]. The Department of Health NHS Reference Costs do not provide a separate code to identify patients on inotropic support. Consequently, we used the code for heart failure or shock instead. This could potentially have led to double-counting for some patients, as this code was also used to identify patients with major complications.

Our model attributed the incremental cost of major complications on the basis of a weighted mean for patients with HRG CC score above the lowest cost category. This assumption is based upon the fact that patients’ postoperative condition should almost always result in their CC score being greater than 3, the upper value defined for most baseline cost categories. The model also assumed that the distribution of severity within complication type e.g. stroke [AA35A–E] for the general inpatient population reflected that for patients post cardiac surgery. To some degree any overestimate of the impact of Floseal upon economic outcomes due to this would be offset by the counter assumption made for minor complications. In this instance, the highest or higher cost category was excluded from the weighted mean calculation, an approach which is also appropriate to reduce potential double counting of costs due to the occurrence of both major and minor complications as a part of the same postoperative clinical syndrome.

The type and distribution of surgical procedures reported by Nasso et al. [8], i.e. coronary 36%, valvular 29%, aortic 17% and combined 18%, is unlikely to precisely reflect the profile for elective primary cardiac surgeries in all UK settings. However, our approach applies the impact of complications and revisions to the observed pattern and costs of surgeries conducted in the UK. Hence, the effect of different surgeries would be upon the nature and type of complications expected and is beyond the scope of our model. Another limitation of our study was that the model inputs for the average number of haemostat kits used per case in the UK was derived from an independent market research study. The sample size of this survey was modest; however, this was the best evidence available to us as no published data were available.

A further important consideration is that the source of clinical inputs to the model was a study involving two comparator agents (Surgicel Nu-Knit and Gelfoam). Gelfoam is not commercially available in the UK; however, the data for these two agents could not be separated, so data for both agents were included in the model. The model therefore depends upon the hypothesis that Surgicel Nu-Knit and Gelfoam deliver equivalent efficacy. There are no clinical studies to support this contention; however, in vitro data [17] suggest that the activity of gelatine-based agents exceeds that of ORC, hence the differences in outcome reported in this model are unlikely to be an overestimate due to this assumption. The patients in whom Gelfoam was used contributed a minority of data (39.8%) to the outcomes used in this model [10]. A recently published cost-consequence analysis reported that the use of Floseal was associated with substantial cost savings for US hospitals compared with another flowable haemostatic agent (SURGIFLO® Hemostatic Matrix Kit, Ethicon–Johnson & Johnson) [18]. The results of this study that used a different comparator agent in a different healthcare setting support the validity of our findings [18].

## Conclusion

The inclusion of NHS-specific healthcare costs into a health economic model based upon the principal clinical and cost inputs associated with the use of haemostatic agents in primary elective cardiac surgery demonstrates significant benefits in favour of the flowable haemostatic matrix (Floseal) over ORC. These benefits commence during the operating theatre and appear to continue to be realised throughout the postoperative period. This model supports the use of Floseal over ORC despite higher acquisition costs.

## Additional file

Additional file 1: Mean number of pieces of haemostat kit used per cardiac surgical procedure (UK data). Market research data that investigated the average number of haemostat kits used in surgical procedures in the UK. (DOC 44 kb)

## Abbreviations

CC: comorbidity and complication
HRG: Health Resource Groups
ICD: International Classification of Diseases
ICU: intensive care unit
LOS: length of hospital stay
NHS: National Health Service
OR: odds ratio
ORC: oxidised regenerated cellulose
UK: United Kingdom
US: United states

## Footnotes

1 SURGICEL® Absorbable Hemostat (oxidized regenerated cellulose) is used adjunctively in surgical procedures to assist in the control of capillary, venous, and small arterial haemorrhage when ligation or other conventional methods of control are impractical or ineffective.
