# Supplementary material for: Use of a flowable haemostat versus an oxidised regenerated cellulose agent in primary elective cardiac surgery: economic impact from a UK healthcare perspective
Source: J Cardiothorac Surg. 2017 Nov 29;12:107. doi: 10.1186/s13019-017-0660-y (PMC5707905; doi:10.1186/s13019-017-0660-y)
Supplement: Additional file 1: — Mean number of pieces of haemostat kit used per cardiac surgical procedure (UK data). Market research data that investigated the average number of haemostat kits used in surgical procedures in the UK. (DOC 44 kb) [file 13019_2017_660_MOESM1_ESM.doc]

## Mean number of pieces of haemostat kit used per cardiac surgical procedure (UK data)

| **Mean number of pieces used by speciality** | **Floseal** | **Surgicel** |
| --- | --- | --- |
| All surgeons, n | 158 | 249 |
| # of pieces | 1.4 | 2.3 |
| Cardiac surgeons, n | 19 | 26 |
| # of pieces | 1.4 | 2.2 |

*Q4b. And please can you indicate – for each of the haemostatic agents and/or tissue sealants shown here – how many pieces / applications do you typically use per procedure, on average?*

*n = number of surgeons asked the question regarding their usage of haemostats in each speciality*

Source: Millward Brown, 2015, Independent Market Research on Haemostasis (funded by Baxter Healthcare).
